# Children, chimpanzees, and bonobos adjust the visibility of their actions for cooperators and competitors

**DOI:** 10.1038/s41598-017-08435-7

**Published:** 2017-08-17

**Authors:** Sebastian Grueneisen, Shona Duguid, Heiko Saur, Michael Tomasello

**Affiliations:** 1 Max Planck Institute for Evolutionary Anthropology, Leipzig, Germany; 2 Duke University, Durham, NC 27708 USA

## Abstract

Chimpanzees and bonobos are highly capable of tracking other’s mental states. It has been proposed, however, that in contrast to humans, chimpanzees are only able to do this in competitive interactions but this has rarely been directly tested. Here, pairs of chimpanzees or bonobos (Study 1) and 4-year-old children (Study 2) were presented with two almost identical tasks differing only regarding the social context. In the cooperation condition, players’ interests were matched: they had to make corresponding choices to be mutually rewarded. To facilitate coordination, subjects should thus make their actions visible to their partner whose view was partially occluded. In the competition condition, players’ interests were directly opposed: the partner tried to match the subject’s choice but subjects were only rewarded if they chose differently, so that they benefited from hiding their actions. The apes successfully adapted their decisions to the social context and their performance was markedly better in the cooperation condition. Children also distinguished between the two contexts, but somewhat surprisingly, performed better in the competitive condition. These findings demonstrate experimentally that chimpanzees and bonobos can take into account what others can see in cooperative interactions. Their social-cognitive skills are thus more flexible than previously assumed.

## Introduction

Chimpanzees (*Pan troglodytes*) are able track what others perceive, know, and intend, and they readily do so to predict other’s actions^1–7^. Although less studied, bonobos (*Pan paniscus*), who shared a common ancestor with chimpanzees about 2 two million years ago, appear to have comparable social-cognitive abilities^8–12^. These abilities, which are key for human forms of cooperation, communication, and culture^13, 14^, thus have deep phylogenetic roots.

However, a striking feature of these studies is that they have mostly used competitive settings: the apes had to use their mental state understanding to gain an advantage over a human or conspecific partner in a contest over food. By comparison, experiments investigating chimpanzee social-cognitive skills in cooperative scenarios have yielded largely negative results^15–18^. This has led to the proposal that due to the competitive nature of their social lives, chimpanzees generally display greater social-cognitive skill in competitive than in cooperative contexts (sometimes called the competitive cognition hypothesis^19–21^). This is in stark contrast to human children who reliably display these abilities in cooperative contexts from early in development^22–24^.

A significant drawback of the competitive cognition account, however, is that chimpanzees have rarely been tested in paradigms that differed only in terms of social context (i.e., cooperation versus competition) but were identical in most other respects^25^. In one of the few exceptions, chimpanzees were presented with an object choice task in which food was hidden underneath one of two cups. In one condition, a cooperative experimenter pointed to the cup containing the food in an attempt to inform subjects of its location whereas in another condition, a competitive experimenter reached for the baited cup and attempted (but failed) to access the food herself. The consistent finding was that chimpanzees only successfully inferred the location of the food in the competitive condition, thus lending some support to the competitive cognition hypothesis^20^ (see also ref. 26).

Understanding this kind of cooperative communicative intention plays a key role in human everyday interaction and comes naturally to human infants^27–29^. However, this is only one specific type of cooperative interaction and the failure of chimpanzees to comprehend altruistically motivated communicative acts, particularly when provided by a human partner, may not be indicative of their skills in cooperative contexts across the board (this is particularly so because chimpanzees are not known to naturally produce such cooperative communicative acts for one another).

A different case of cooperative interaction in humans is one in which multiple individuals work together collaboratively and mutualistically, as when collectively foraging or building things together^13, 30, 31^. Chimpanzees and bonobos also engage in mutually beneficial collaborative activities in their natural habitat as, for example, when hunting for monkeys or teaming up with partners during conflicts^32–36^ and this has been demonstrated in experimental contexts as well^37–39^. An important difference is that these interactions do not necessarily require individuals to ascribe benevolent intentions to others. Instead, and similarly to competitive scenarios, it is sufficient to assume self-serving intentions or to simply project one’s own preferences onto others (e.g., just like myself, my partner wants to catch the monkey). Taking into account other’s mental states seems particularly relevant to making good decisions in mutualistic contexts (e.g., Does my partner know where the monkey is? Which monkey can my partner see?), so that, perhaps, apes are more likely to display social-cognitive abilities in this kind of interaction. Whether chimpanzees and bonobos are able to do this or if these skills are indeed restricted to competitive settings is presently unresolved.

**Figure 1:**
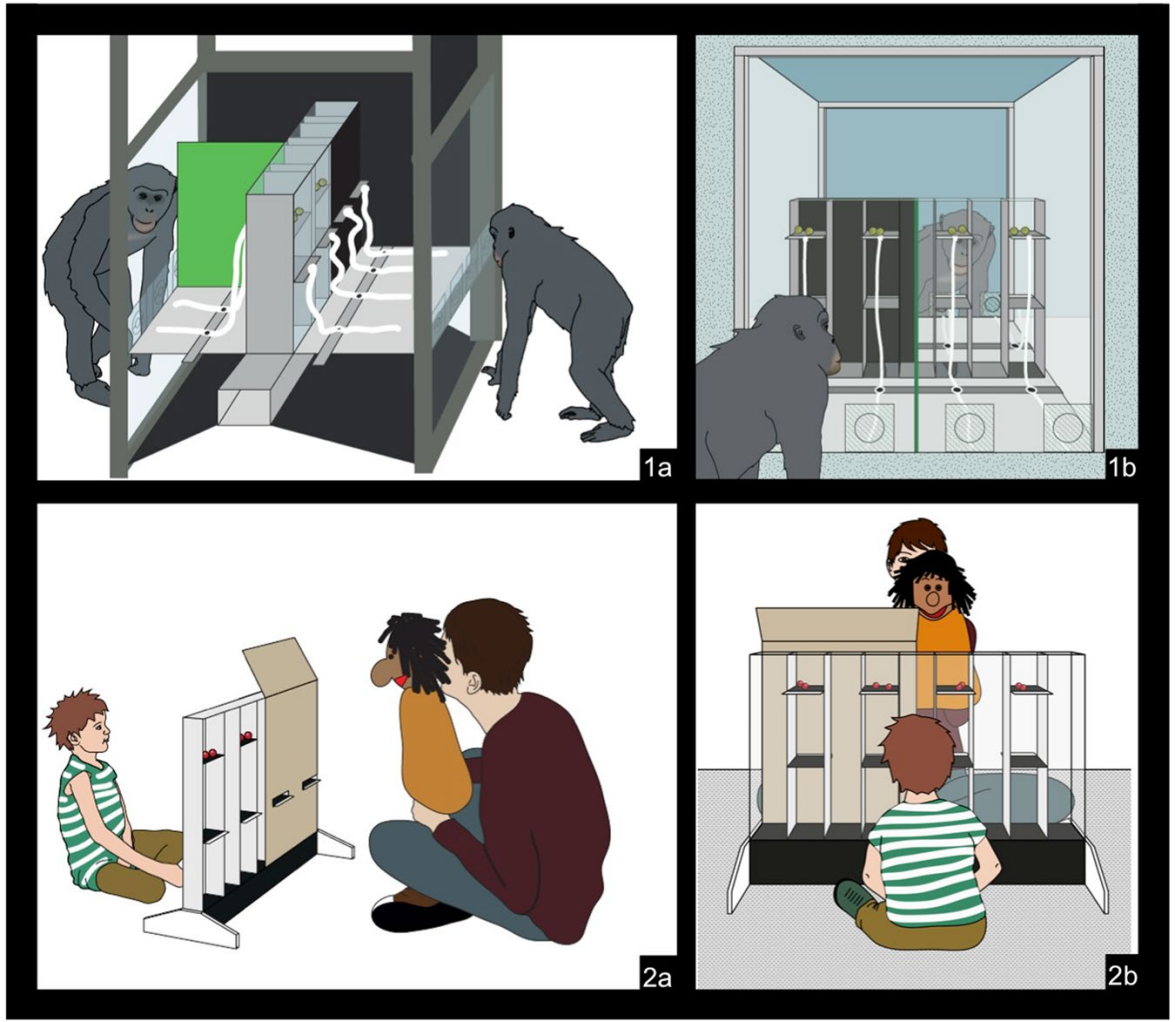
Experimental setup. 1a) Setup of Study 1 – the subject (left) chooses first, the stooge partner (right) chooses second; 1b) Apparatus from the subject’s perspective – subjects can see the whole apparatus, the partner’s view is partially blocked by a barrier; 2a) Setup of Study 2 – children choose first, the partner (a puppet played by E2) chooses second; 2b) Apparatus from the child’s perspective, the puppet’s view is partially blocked by a barrier. (copyright holder: Max Planck Society).

The current study attempted to fill this gap. In an experimental setting, pairs of apes (and, in a second study, human children) were presented with an apparatus in which they could either hide or display their actions to their partner whose visual access to the apparatus was partially blocked by a barrier (Fig. 1). In the cooperation condition, both players had identical interests: by choosing to act on the same part of the apparatus they received one reward each. To facilitate coordination, subjects should therefore choose a part of the apparatus visible to their partner. In the competition condition, in contrast, players’ interests were directly opposed: the partner still tried to match the subject’s choice, but the subject only received a reward by choosing a different part of the apparatus from their partner. They should therefore choose a part of the apparatus not visible to their partner. In both conditions, therefore, subjects could benefit from taking into account what their partner could see, the difference being that in one condition they cooperated mutualistically while in the other condition they competed.

To increase the external validity of the study, subjects interacted with conspecific partners rather than human experimenters. If apes are indeed better at applying their social-cognitive skills in competitive settings, they should perform better in the competitive than in the cooperative condition. By contrast, if they can apply their skills in both contexts equally, and failure in previous experiments was due to the specific kind of cooperative activity, subjects should succeed in both tasks. For comparison, we also tested preschool children in a second study using analogous tasks (Study 2). Since preschoolers have previously been shown to understand what others can see we predicted children to successfully adapt their decisions to the social context and to perform well in both tasks.

## Results

### Study 1a (between-subjects)

A Generalized Linear Mixed Model (GLMM) revealed that the apes chose the visible option significantly more often in the cooperation condition than in the competition condition (χ^2^ = 4.44, df = 1, *p* = 0.035) providing a first indicator that they adjusted the visibility of their choices depending on the social context in the direction predicted. The model controlled for species (chimpanzee vs. bonobo), barrier position, session and trial number, the random effects of subject ID and the social group, and the random slopes of barrier position, session, and trial number nested within subject ID (see Method section and Supplementary Information for details on these predictors and Supplementary Tables S1 and S2 for detailed model descriptions and outputs).

**Figure 2:**
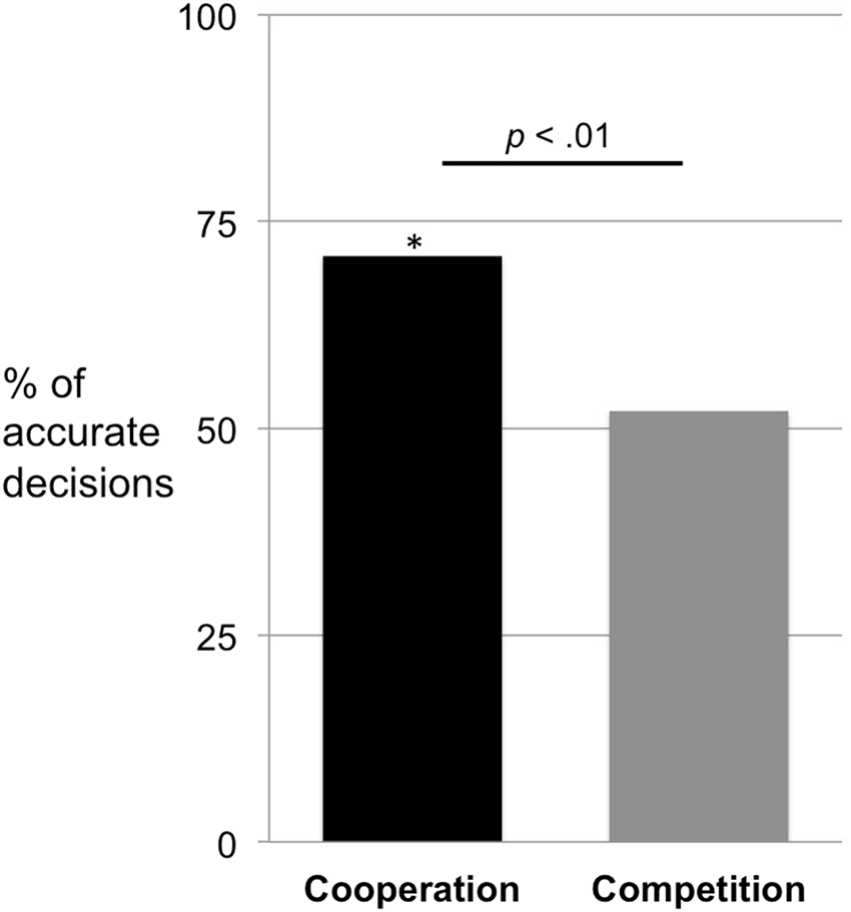
Study 1a – % of accurate decisions – i.e. visible decisions in the cooperation condition and hidden decisions in the competition condition – by chimpanzees and bonobos (out of 24 trials). *Above chance (p < 0.05).

A second GLMM examined subject’s tendency to make accurate decisions (i.e. visible choices in the cooperation condition and hidden choices in the competition condition). Subjects in the cooperation condition made significantly more accurate decisions than subjects in the competition condition (χ^2^ = 7.14, df = 1, *p* = 0.008; Fig. 2) and chimpanzees made more accurate decisions than bonobos (χ^2^ = 4.21, df = 1, *p* = 0.040). The model included the same predictors, random effects, and random slopes as the first GLMM, with the only difference that species was treated as a test predictor to investigate potential species differences in task performance.

Further analyses revealed that subjects chose the correct option significantly above chance (i.e., 50%) in the cooperation condition (mean = 17 (70.8%), SD = 3.35, *t*(5) = 3.66, *p* = 0.015) but not in competition condition (mean = 12.5 (52.1%), SD = 3.78, *t*(5) = 0.32, *p* = 0.759). Session or trial number did not have a significant effect in any of the models.

### Study 1b (within-subjects)

Study 1a provided first strong experimental evidence that chimpanzees and, to a lesser degree, bonobos are able to take into account what others can see in cooperative interactions. These skills thus do not appear to be restricted to competitive contexts per se. A surprising finding, however, was that subjects actually performed better in the cooperative than in the competitive condition. To further examine the flexibility of the apes’ abilities and to reduce the likelihood that these results were influenced by random assignment we presented subjects with the identical experiment again, except that individuals who had previously taken part in the cooperation condition now participated in the competition condition (and vice versa).

To analyze the complete dataset, we ran the same two GLMMs as in Study 1a except that we included the experimental phase and the interaction between condition and phase as additional test predictors to detect potential order effects. The interaction between condition and phase did not significantly affect subjects’ tendency to make their choices visible (χ^2^ = 0.02, df = 1, *p* = 0.885) and was therefore dropped from the model. The main effect of phase was also not significant (χ^2^ = 1.31, df = 1, *p* = 0.252). However, and confirming the results of Study 1a, apes chose the visible option significantly more often in the cooperation condition than in the competition condition (χ^2^ = 9.11, df = 1, *p* = 0.003).

**Figure 3:**
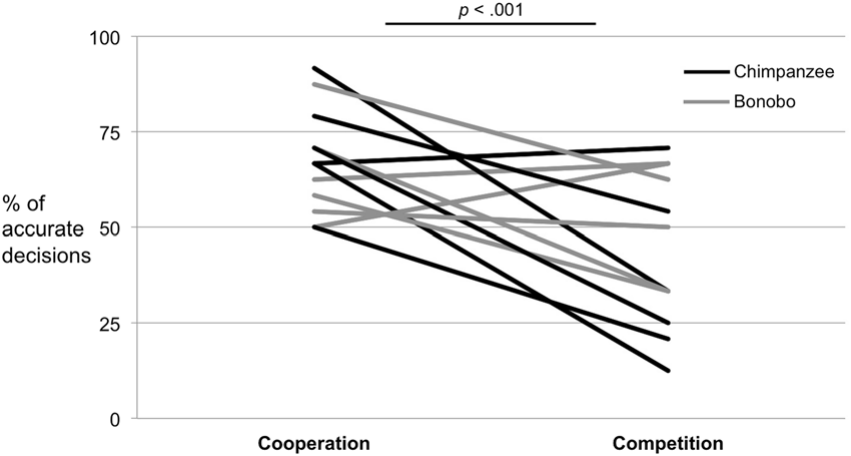
Study 1b – % of accurate decisions – i.e. visible decisions in the cooperation condition and hidden decisions in the competition condition – by individual chimpanzees and bonobos (out of 24 trials per condition).

In the GLMM analyzing decision accuracy the interaction between phase and condition was also not significantly (χ^2^ = 1.59, df = 1, *p* = 0.207) and was thus dropped from the model. However, subjects made significantly more accurate decisions in the cooperation than in the competition condition (χ^2^ = 37.40, df = 1, *p* < 0.001), thus confirming the result of Study 1a (Fig. 3). The species difference found in Study 1a was not confirmed (χ^2^ = 0.21, df = 1, *p* = 0.645). Moreover, subjects made significantly more accurate decisions in phase 1 than in phase 2 of the experiment (χ^2^ = 8.47, df = 1, *p* = 0.004).

Further analyses revealed that, overall, subjects chose the correct option significantly above chance in the cooperation condition (mean = 16.25 (67.7%), SD = 3.25, *t*(11) = 4.53, *p* = 0.001) but not in competition condition (mean = 10.58 (44.1%), SD = 4.83, *t*(11) = −1.02, *p* = 0.332). Again, session or trial number did not have a significant effect in any of the models.

### Follow-up preference test

To further investigate the result that subjects made more correct choices in the cooperation condition than in the competition condition we ran an additional preference test to assess whether the apes might have a baseline preference for making visible or hidden choices in the presence of a partner in a neutral context. Subjects (including individuals who had not participated in the main experiment) could choose one of four baited trays, two of which were occluded by a barrier from a partner’s view (as in the main experiment). The partner remained passive and could not access any food.

Subjects did not show a preference for visible trays: choices were not different from chance (mean = 12.45 (51.9%), SD = 2.58, *t*(19) = 0.81, *p* = 0.427) suggesting that the difference in performance in the main conditions is unlikely to be due to a baseline preference for the visible options (there was no significant difference between subjects who had taken part in the main experiment and those who had not, *t*(18) = 0, *p* = 1).

### Study 2

For comparison, we ran an analogous study with 4-year-old children. This age group was chosen since previous research has demonstrated first competencies at this age in other cooperative coordination problems^40, 41^. While previous studies have shown that visual perspective taking skills are present by four years of age^42, 43^ it has rarely been examined in how far children can apply these skills in strategic interactions. Moreover, and similar to research with apes, this has not been studied in tasks comparable in their overall structure but varying in terms of the social context. Children were thus presented with almost identical tasks as the apes (with only minor adjustments, see Method section) in a between-subjects design.

### Choices

**Figure 4:**
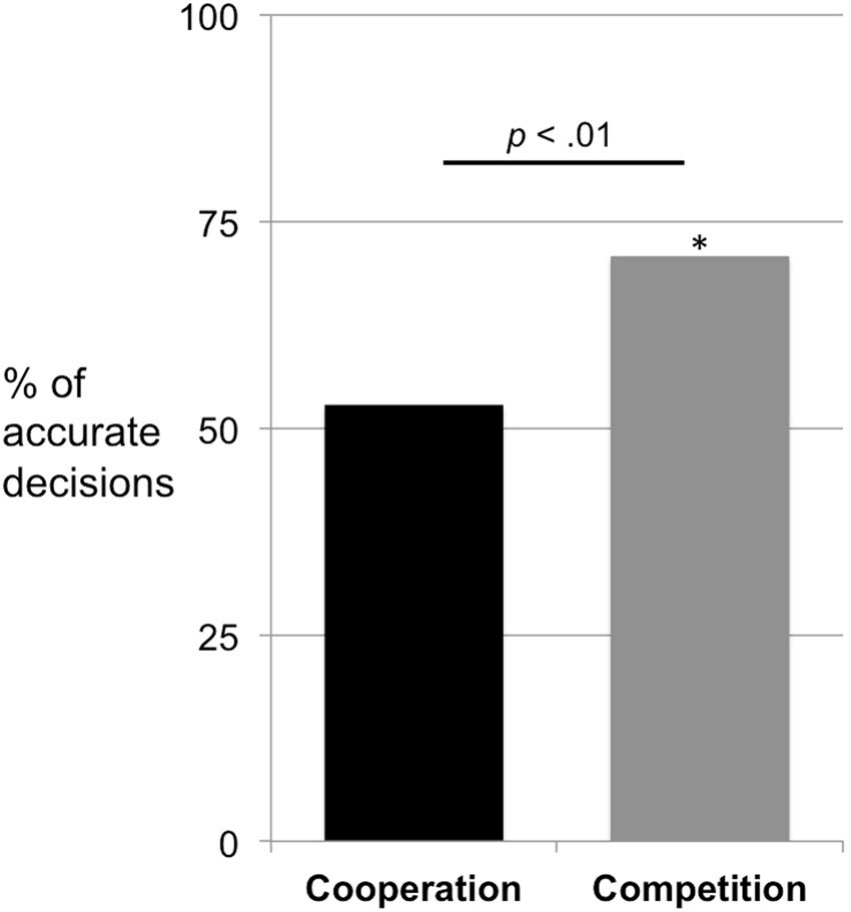
Study 2 – % of accurate decisions – i.e. visible decisions in the cooperation condition and hidden decisions in the competition condition – by children (out of 6 trials). *Above chance (p < 0.01).

To analyze children’s choices we ran a GLMM with condition as the only test predictor while controlling for trial number, identity of the second experimenter, barrier position, the random effect of subject ID, and the random slopes of trial number and barrier position nested within subject ID (see Method section and Supplementary Information for details on the predictors and Supplementary Tables S3 and S4 for detailed model descriptions and outputs). In line with our predictions, children chose the visible option significantly more often in the cooperation condition than in the competition condition (χ^2^ = 9.37, df = 1, *p* = 0.002; Fig. 4). A second GLMM (using the same test and control predictors) indicated that children made fewer correct choices in the cooperation than in the competition condition (χ^2^ = 9.61, df = 1, *p* = 0.002). Further analyses showed that children chose the correct option above chance in the competition condition (mean = 4.25 (70.8%), SD = 1.70, *t*(23) = 3.44, *p* = 0.002) but not in cooperation condition (mean = 3.17 (52.8%), SD = 1.66, *t*(23) = 0.492, *p* = 0.627).

### Post-test questions

As an additional measure of children’s understanding of the task they were asked after the test: “When was it easier for you to win the marbles – when you pulled behind the barrier or when you pulled where there was no barrier?” Children in the cooperation condition were more likely to indicate the visible options than children in the competition condition (Fisher’s Exact Test, *p* = 0.005). The number of accurate decisions children made at test was positively related to the accuracy of children’s responses (i.e. to refer to the visible options in the cooperation condition and to the hidden options in the competition condition; GLM (Generalized Linear Model): *χ* ^2^ = 4.71, df = 1, *p* = 0.030). Moreover, children in the cooperation condition provided more accurate answers than children in the competition condition (χ^2^ = 5.86, df = 1, *p* = 0.015), which contrasts with their decisions at test where they were more accurate in the competition condition.

Children were then asked: “Why was it was easier to win marbles there?” A GLM indicated that the number of accurate decisions at test was positively related to children’s tendency to refer to their partner’s perceptual or knowledge state (e.g. “because she could see the marbles there” after indicating the visible options in the cooperation condition, or “because she didn’t know which tower I chose” after indicating the hidden options in the competition condition; χ^2^ = 12.77, df = 1, p < 0.001). The experimental condition did not significantly affect the accuracy of children’s responses (χ^2^ = 0.29, df = 1, p = 0.590).

### Follow-up preference test

A similar preference test as with the apes was run with a new sample of children. The apparatus was rigged so that children were able to retrieve rewards individually while the partner remained passive. The training and test mimicked the main experiment as closely as possible. Children’s choices in the preference test were not different from chance (mean = 3.13 (52.2%), SD = 1.36, *t*(23) = 0.45, *p* = 0.657) suggesting that children’s choices in the test conditions were not influenced by a baseline preference for hidden or visible options.

## Discussion

Our findings indicate that the ability of chimpanzees and bonobos to recruit their social-cognitive skills is not restricted to competitive interactions: apes in the current study successfully adjusted the visibility of their decisions depending on whether they cooperated or competed with a partner, and strikingly, they performed particularly well in the cooperative task.

This is in stark contrast to previous studies investigating chimpanzee social-cognitive skills in cooperative interactions^15–18^. Unlike the current study, however, past research largely investigated apes’ abilities in the context of altruistic communication. For example, chimpanzees have been shown to fail to reliably retrieve food hidden underneath one of two cups even though an experimenter had previously pointed to the baited cup^20^. Crucially, subjects in this task were not only required to comprehend that the experimenter knew about the location of the food but also that she had the benevolent intention to inform subjects of its whereabouts – something that does not appear to occur naturally among chimpanzees.

By contrast, the mutualistic context of the current study meant that subjects did not have to ascribe intentions to their partner that differed from their own. Instead, and just as in the competitive condition (as well as in competitive tasks used by Hare and colleagues^1^), they only had to assume that, like themselves, their partner intended to access the food. This may have enabled them to treat their partner as a ‘social tool’ that they could use to reach an individualistic goal^44^. Moreover, chimpanzees and bonobos have been shown to cooperate mutualistically in the wild and in laboratory settings so that this may be a context in which they are naturally capable of displaying their social-cognitive abilities. An additional advantage of the current study was that subjects interacted with conspecific partners rather than human experimenters which added to the external validity. The absence of an effect of session or trial number and the observation that performance did not drop when the barrier position changed further suggests that the current findings cannot be explained by learning during the test sessions.

Unexpectedly, however, subjects’ performance was better in the cooperative than in the competitive condition which conflicts with previous studies using competitive scenarios^1–7^. Why the apes failed to adjust the visibility of their choices in the competitive task of the current study is not entirely clear. One possibility, that the apes have a baseline preference for the visible options, was refuted by the absence of such a preference in the post-test preference test. An alternative is that even though the apes received extensive experience with the competitive context at training and successfully completed all criterion tests subjects may have understood the setup of cooperative task better than the setup of the competitive task. The possibility that the observed difference between conditions was due to differences in task understanding thus cannot be fully excluded.

It is important to note, however, that some previous studies examining chimpanzees’ social-cognitive skills in competitive contexts have also yielded negative results. For instance, in a recent study by Karg and colleagues chimpanzees successfully revealed food to a helpful experimenter who gave them any visible food (although they did so rarely before the experimenter approached in anticipation of her actions) but failed to manipulate the same apparatus such that food was concealed from a competitor’s view^45^. A parallel between this study and the current one is that in contrast to previous studies^1–3^ subjects were not required to hide themselves or to consider whether or not another individual had seen some food being hidden but to directly manipulate the rewards while anticipating what their partner would see when it was their turn to choose. This may have added complexity in both this and the current tasks. Given the wealth of evidence showing chimpanzees’ abilities to use their social-cognitive skills in competitive interactions the findings of the current studies should not be interpreted as suggesting that chimpanzees are better at using these skills in cooperative than in competitive interactions. They do suggest, however, that their abilities to use these skills may be influenced by contextual factors that are not yet fully understood and require further investigation.

Importantly, the current findings provide some first strong experimental evidence that chimpanzees and bonobos are able to use their social-cognitive abilities in order to successfully coordinate decisions with conspecifics in a cooperative context. Contrary to previous suggestions^19–21^, these abilities may thus not be restricted to competitive interactions. This corresponds to the recent finding that wild chimpanzees selectively inform ignorant group members of danger^46^ which also points to the conclusion that chimpanzees can recruit their understanding of other’s mental states more flexibly and across social contexts.

In the second study, 4-year-old children also successfully adapted their decisions to the social context and chose the visible option significantly more often in the cooperation condition than in the competition condition. The significant relation between children’s performance at test and their ability to correctly respond to the post-test questions (i.e., their verbal task understanding and the tendency to explicitly refer to their partner’s perception or knowledge) further underlines that children did indeed solve the task by taking into account their partner’s mental states.

Somewhat surprisingly, however, children performed better in the competition condition than in the cooperation condition and, as with the apes, this finding is unlikely to be accounted for by a baseline preference for hidden or visible options as indicated by the follow-up preference test. In contrast, previous research has shown that children at this age can take into account what others can see and how this differs from their own perspective^42, 43, 47^ and also readily recruit communication and complex theory of mind skills for cooperative purposes^40, 41, 48^.

While unexpected, several reasons may account for this finding. First, many games that children encounter in their daily lives involve some form of competition^49^ so that children may find it more intuitive to compete with others in social game contexts. Relatedly, the partner’s motive to steal marbles may have been particularly salient, leading children to be more attuned to the test situation in the competitive condition.

On the other hand, children in the cooperation condition were very capable of accurately answering the post-test questions suggesting that the discrepancy in performance between conditions may have been due to differences in motivation rather than comprehension. Thus, another possibility is that children did understand that choosing the visible options always resulted in a certain outcome (i.e., always winning in the cooperation condition and always losing in the competition condition) and that hiding their decision made the game more exciting, despite the fact that this resulted in lower rates of overall success in the cooperation condition. The introduction of new rewards at test (golden marbles, see method section) was an attempt to prevent such alternative motives but the possibility that choosing behind the barrier in the cooperation condition may simply have been more fun cannot be fully excluded.

Finally, previous research has shown that social engagement can sometimes lead children to overestimate other’s knowledge^50^. In this study, 2-year-olds played with an object an adult could not see. When the adult remained co-present – but not when she disengaged from the interaction – children often erroneously assumed the adult to be acquainted with the object. Something similar may potentially have been at play in the current experiment although it is unclear why this should have exclusively affected the cooperation condition. What speaks in favor of this explanation, however, is that, on a few occasions, children in the cooperation condition chose an option behind the barrier and then – invisible to the partner – pointed to that option as if attempting to inform the partner which tower to choose. Hence, given that children could always make mutual eye contact and communicate with their partner some participants may have assumed their own decisions to be mutually known, a possibility worth investigating further in future experiments.

In conclusion, the current study demonstrates that chimpanzees and bonobos are able to recruit their understanding of other’s minds more flexibly than commonly thought. Whereas these abilities have been suggested to be largely restricted to competitive settings the current investigation shows experimentally that chimpanzees and bonobos are also able to take into account what others can see in order to cooperate with conspecifics for mutual benefit. This attests to chimpanzees’ and bonobos’ cooperative abilities in mutualistic contexts. The study further highlights the importance of investigating the influence of the social context on the use of social-cognitive skills in humans and other apes by using comparable experimental paradigms.

## Method

### Study 1a

#### Subjects

Eight chimpanzees (*Pan troglodytes*, 3 females, *M*_age_ = 14 years) from two different social groups and 4 bonobos (*Pan paniscus*, 3 females, *M*_age_ = 16 years) were included in the final sample (for a full subject list see Supplementary Table S5). Five additional chimpanzees and three bonobos did not complete the training and were not included in the test. Two further chimpanzees and one bonobo acted as stooge partners. All were housed in social groups in at the Wolfgang Köhler Primate Research Center. Subjects were randomly allocated to the cooperation or the competition condition (between-subjects design) with the constraint that per condition there was an equal number from each species.

The study complied with the European and World Associations of Zoos and Aquariums (EAZA and WAZA) Ethical Guidelines and was approved by the joint Ethics Committee of Leipzig Zoo and the Max Planck Institute for Evolutionary Anthropology. Subjects were never food-deprived and water was available throughout testing. Participation was voluntary and could be refused at any time.

#### Apparatus and design

The test apparatus was a transparent plexi-glass box including four adjacent towers (45 cm high, 6 cm wide, with 8 cm space between them). Each tower contained two platforms – an upper and a lower one – and ropes were attached to the platforms. The “proposer” could access the ropes attached to the upper platforms and the “responder” the ones attached to the lower platforms (proposer and responder were in adjacent rooms). Two rewards were placed on each upper platform. To access a rope subjects had to lift a decision door in front of that rope. This ensured that they could not pull more than one rope simultaneously. Once subjects had made a choice (i.e. pulled one rope) the experimenter moved the other ropes out of reach.

In each trial, the proposer started by pulling a rope, causing the corresponding platform to collapse and the rewards to fall down onto the lower platform. The responder then collapsed a lower platform. Reward dispersal was controlled via a series of plastic ramps invisible to the apes and worked differently depending on the condition. In the cooperation condition, rewards ending up at the bottom of the towers were divided equally between proposer and responder. Hence, if both collapsed platforms of the same tower they each received one reward. If they chose different towers no player could access the rewards.

In the competition condition, the responder had exclusive access to all rewards at the bottom of the towers so that by choosing the same tower as the proposer the responder could steal all rewards. The proposer received all rewards remaining on the lower platforms. That is, if the responder chose a different tower than the proposer the experimenter waited a few seconds, switched the position of the ramps, and collapsed the lower platforms so that all rewards of the tower the subject had chosen rolled to the subject’s side while the partner received nothing. Hence, in the cooperation condition players were incentivized to coordinate on the same tower. In the competition condition, the responder’s goal was to collapse the platform of the same tower as the proposer but the proposer’s goal was for both to choose different towers.

Rewards were banana pellets for all but one chimpanzee and grapes for the bonobos and the remaining chimpanzee (who was unmotivated by pellets).

### Procedure

#### Training

Subjects completed several training phases to ensure thorough comprehension of the test situation. First, they learned how to access the ropes attached to the platforms (door training) after which they were introduced to the full apparatus and the reward dispersal of the corresponding experimental condition (apparatus familiarization). In both conditions, subjects then had to demonstrate apparatus understanding by completing two criterion tests, once by successfully responding to a partner’s choices (responder training) and once by operating the apparatus alone on both sides (open-door training). Only subjects who reached criterion proceeded to the test. Finally, subjects in both conditions received experience on both sides of the apparatus with a barrier occluding the responder’s view of all towers (barrier experience). This was done to familiarize subjects with the barrier and for them to experience how the barrier obstructed one’s view in the responder position (in this phase the apparatus was rigged so that in both conditions subjects were rewarded equally often in the presence of a barrier prior to test). For details on all training steps and criterion tests see ‘detailed training procedures’ in Supplementary Information.

#### Test

Subjects acted as the proposer and a conspecific partner as responder. A barrier occluded the responder’s view of two towers. Subjects could see all four towers (see Fig. 1 for view from subject’s perspective). In addition to completing the training phases partners received additional experience with the barrier to ensure they responded in ways that maximized their food intake – i.e. to match the subject’s choice if the subject had chosen a visible option and to choose randomly between the hidden options if the subject had chosen behind the barrier (this was the same in both conditions – the partner’s aim was always to match the subject’s choice whereas whether or not the subject was incentivized to facilitate a match depended on the condition). Partners performed their task very reliably and made only few mistakes so that, overall, they successfully matched the subject’s choice in 98.0% of trials when subjects had chosen a visible option and in 44.6% of trials (i.e. close to the 50% chance level) when subjects had chosen an option behind the barrier.

The test consisted of two sessions of 12 trials (for a total of 24 trials), with the barrier position being swapped halfway through each session. Which towers were covered first was counterbalanced across subjects.

### Study 1b

Training and test was identical to Study 1a except that subjects who had previously taken part in the cooperation condition participated in the competition condition (and vice versa) in a within-subjects design.

### Follow-up preference test

#### Subjects

Sixteen chimpanzees (*Pan troglodytes*, 9 females, *M*_age_ = 25 years) and 4 bonobos (*Pan paniscus*, 3 females, *M*_age_ = 15 years) participated. Three further chimpanzees and one bonobo took part as stooge partners. Eight chimpanzees and three bonobos had also taken part in Study 1.

#### Apparatus

The apparatus consisted of four plastic trays (8 × 8 cm), each with a string attached. Trays each contained two rewards and were placed in the same position as the towers in the main experiment. Subjects could access the food in the proposer position by pulling on the strings. Partners in the responder position could not access any food and remained passive.

### Procedure

#### Training

Before the test subjects demonstrated that they understood how to access the trays and received experience with a full barrier as in Study 1 (see Supplementary Information).

#### Test

Subjects were in the proposer and the partner in the responder position. A barrier occluded the partner’s view of two trays while all trays were visible to the subject. The barrier position was switched halfway through the session and was counterbalanced in the same way as in Study 1. Subjects completed two sessions of 12 trials.

### Study 2

#### Participants

Forty-eight 4-year-olds (*M*_age_ = 4.55 years, 50% girls) participated in the study. Children mostly came from middle class backgrounds and were recruited and tested at urban daycare centers. One additional child failed to pass the training criteria and was excluded from the test. Children were randomly assigned to the cooperation and competition conditions in a between-subjects design.

The study was conducted in compliance with the ethical principles of the American Psychological Association (APA) and was approved by the internal Ethics Committee of the Max Planck Institute for Evolutionary Anthropology. Children’s participation occurred with their parent’s informed consent, was voluntary, and could be refused or terminated at any time.

### Apparatus and design

The general task was the same as for the apes except for the following changes. Instead of operating decision doors and pulling on ropes, children pulled out the platforms directly and were told they could only make one choice per round. In the cooperation condition, a sliding door was in the bottom of the apparatus on both sides. At the end of each round, the experimenter opened the sliding doors and – if players had coordinated successfully – each player could retrieve one reward (otherwise both received nothing).

In the competition condition, the sliding door on the proposer side was moved up to the level of the lower platforms (for the responder it remained at the bottom). The responder could thus access all rewards at the bottom of the apparatus whereas the proposer had exclusive access to all rewards remaining on the lower platforms resulting in the same payoff structure as in Study 1 (Fig. 1).

Red and golden marbles were used as rewards at training and test, respectively.

### Procedure

#### Training

The training closely resembled that of Study 1. Children were first familiarized with the apparatus (apparatus familiarization) and introduced to the partner (a same-sex puppet played by a second experimenter). Children then completed two criterion tests (responder training and open-door training) to demonstrate apparatus understanding and gained experience with a barrier covering the all towers from the responder’s perspective (barrier experience). As in Study 1, rewards were dispersed during training according to the experimental condition to familiarize participants to the respective social context (i.e. cooperation versus competition). To accommodate the constraint that testing had to be completed within one day, sessions were shortened and some minor training features replaced by verbal instructions (see Supplementary Information).

#### Test

The general test was the same as for the apes. To keep motivation high, golden marbles were introduced as rewards. The test consisted of six trials and after three trials the position of the barrier was swapped (the starting position was counterbalanced). As in Study 1, the partner chose in a way that maximized her payoff. That is, in both conditions the partner matched children’s choices if they chose a visible option and chose randomly between the two hidden options if children chose behind the barrier.

After completing the test children were asked the two post-test questions after which they made a necklace with the marbles collected during the game.

### Follow-up preference test

#### Participants

Twenty-four 4-year-olds (*M*_age_ = 4.56 years, 50% girls) participated.

#### Apparatus

The apparatus was the same as in Study 2 except that the lower platforms were removed so that participants could release the rewards alone. Children played with the same puppet as in Study 2.

### Procedure

#### Training

Before test, children successfully retrieved the rewards in a training procedure closely matching that of Study 2. As in Study 2, children also received experience with a full barrier (see Supplementary Information).

#### Test

A barrier occluded the partner’s view of two towers while all towers were visible to the subject. Children completed six trials. The barrier position was switched halfway through the session.

#### Coding

For each trial of Studies 1 and 2, we coded whether subjects chose a tower behind the barrier (hidden) or a tower the responder could see (visible) and whether or not this choice was the right one (i.e. visible in the cooperation condition and hidden in the competition condition).

Children’s responses to the firs post-test question were coded as referring to visible or hidden towers, and as correct (referring to visible towers in the cooperation condition and to hidden towers in the competition condition) or incorrect. Responses to the second question were coded as referring to the responder’s perceptual or knowledge state or not.

Choices in the follow-up preference tests were coded as visible or hidden.

### Analysis

All models were fitted in R^51^ using the functions ‘glm’ (for Generalized linear models) and ‘glmer’ (for Generalized Linear Mixed Models) of the R-package lme4^52^. For all models, we ran several model diagnostics. These were all unproblematic. If there were multiple test predictors we always first compared a full model with a null model not including the test predictors but retaining all control predictors, random intercepts, and random slopes. We only tested for potential effects of individual test predictors if the full-null model comparison indicated that all test predictors combined significantly affected the response. For detailed model descriptions and outputs see Supplementary Tables 2–5.

## Electronic supplementary material

Supplementary material
