# Supplementary material for: Children, chimpanzees, and bonobos adjust the visibility of their actions for cooperators and competitors
Source: Sci Rep. 2017 Aug 17;7:8504. doi: 10.1038/s41598-017-08435-7 (PMC5561202; doi:10.1038/s41598-017-08435-7)
Supplement: Supplementary file 1 — Supplementary material [file 41598_2017_8435_MOESM1_ESM.pdf]

Children, chimpanzees, and bonobos adjust the visibility of their actions for cooperators and competitors

Sebastian Grueneisen, Shona Duguid, Heiko Saur, & Michael Tomasello

Supplementary Information

Table of contents

Detailed training procedures
a) Study 1 – Apes
b) Study 2 – Children

Statistical analyses
a) General information
b) Study 1 – Apes
    Table S1 – Model descriptions
    Table S2 – Detailed model outputs
c) Study 2 – Children
    Table S3 – Model descriptions
    Table S4 – Detailed model outputs

Table S5 – Ape subject overview

Detailed training procedures

a) Study 1 – Apes

Main experiment

Door training

To access the ropes subjects had to lift small transparent plastic doors. To familiarize subjects with this mechanism they had to retrieve a piece of food from behind each of the doors. One chimpanzee failed to do this and did not take part in the study. All others succeeded within one session.

Apparatus familiarization

Subjects were introduced to the test apparatus. Subjects assumed the proposer role and an experimenter acted as the responder (one session, 12 trials). All towers were baited on each trial. The experimenter chose the same tower as the subject in 9/12 trials. In the remaining trials she chose one of the other towers at random (the responder thus mostly acted as a self-interested individual would while making a few 'mistakes'). Reward dispersal worked according to the experimental condition so that in the cooperation condition both the subject and the human responder received a reward when the responder matched the subject's choice and both received nothing if responder chose a different tower than the subject. In the competition condition, the responder received two rewards by matching the subject's choice while the subject received nothing. If the responder failed to match the subject's choice the subject received two rewards and the responder received nothing. This phase thus gave subjects experience with the cooperative or the competitive context (depending on the condition they were assigned to).

Apparatus understanding

Subjects acted as responder and were required to choose the same tower as the proposer (experimenter) to receive food (reward dispersal worked according to the respective experimental condition). Each session consisted of 12 trials. The experimenter chose each of the four towers three times in a predetermined, randomised order. Subjects had to choose the correct tower significantly above chance (10/12) in two consecutive sessions which took them 4.3 sessions on average (range: 2-7). Three chimpanzees and three bonobos did not reach criterion within 8 sessions and were excluded from the study. This phase further familiarized subjects with the social context of their condition (i.e. cooperation versus competition) and

they learned that the responder was always incentivized to choose the same tower as the proposer.

Open-door training

Only two of the four towers were baited (randomly selected on each trial) and the door between the two rooms was open so that subjects had access to the apparatus from both sides. The ropes on the proposer side were moved into reach first, and once subjects had made a choice they moved around and made a choice on the responder side. In the cooperation condition subjects could then collect the rewards on both sides of the apparatus. In the competition they could only collect the rewards on the responder's side. To enter the next phase, subjects had to successfully access the food by choosing corresponding towers in at least 10 out of 12 trials in two consecutive sessions which took them 3.6. sessions on average (range: 2-7). One chimpanzee did not reach criterion within 8 sessions and were excluded from the study. This phase emphasized the interdependence between proposer and responder (i.e. that choices had to be made on both sides). They also learned that choosing the same tower would only lead to rewards being released on both sides in the cooperation condition.

Barrier experience

A barrier spanning the whole apparatus prevented the responder from seeing the proposer's choice (the proposer's visual access remained unobstructed). Through slits in the barrier the responder could still pull the ropes and collapse any of the lower platforms. At the start of each test session subjects received two trials in the responder role followed by two trials in the proposer role. The partner was the same conspecific stooge as in the following test trials. Trials were rigged so that subjects were successful once in each role in both conditions. Hence, in both conditions subjects were rewarded equally often in the presence of a barrier before entering the test. These experience trials familiarized subjects with the barrier and showed them how the barrier obstructed the responder's view of the apparatus. They also highlighted that all four options could still be chosen in the presence of the barrier.

Follow-up preference tests

Apparatus understanding

One of the trays was baited and subjects had to obtain the food significantly above chance in two consecutive sessions (10/12 trials). All subjects succeeded within two sessions.

Barrier experience

A stooge partner was in the responder position. All trays were baited and subjects chose one tray on four trials, twice without a barrier and twice in the presence of a full barrier spanning the whole apparatus and blocking the stooge's view of the trays. Subjects and stooge then switched positions and the stooge chose four times while the subject remained passive (twice with and twice without the barrier). This was completed at the start of each test session.

b) Study 2 – Children

Main experiment

Apparatus familiarization

An experimenter (E1) explained to children (C) that the aim of the game was to win as many marbles as possible. The partner was a same-sex puppet played by a second experimenter (E2; the E2 had to be replaced halfway through data collection. Each E2 tested the same number of children of each sex per condition). All towers were baited with red marbles. C were in the proposer and the puppet in the responder role. Depending on the condition, E1 emphasized the cooperative or competitive nature of the game ("You can win the marbles together." vs. "Max/Lola can try to steel the marbles."). On the first trial the puppet chose a different tower than C. E1 then opened the sliding doors while saying "round is over." The puppet exclaimed "Oh no, I made a mistake, this time, we won't get the marbles/I won't get the marbles" (cooperation/competition condition, respectively). E1 reset the apparatus and the trial was repeated twice except that now the puppet chose the same tower as C. In all subsequent trials the puppet expressed joy ("Yes, this time we/I win the marbles") or disappointment ("On no, this time we/I won't win any marbles") depending on the outcome of the round. In the cooperation condition the child and the puppet collected their marbles together in a joint container whereas they received separate containers in the competition condition. While C and the puppet made their choices E1 always turned away and pretended to be busy with something else.

Apparatus understanding

C assumed the responder and the puppet the proposer role. If C chose the correct platform (i.e. of the same tower as the puppet) in three out of four trials they entered the next training phase. If not, they were excluded from the experiment. Only one child failed to pass criterion and – apart from one other child – all children completed this phase without mistakes.

Open-door

Two of the four towers were baited (randomly selected on each trial) and C had to operate the apparatus alone by collapsing corresponding platforms on both sides. To enter the final training phase, they had to successfully retrieve the rewards on three of four trials. All children passed without mistakes.

Barrier experience trials

The setup was the same as for the apes. C first received two trials as responder and then two trials as proposer. These experience trials familiarized C with the barrier while highlighting that all four options could still be chosen in the presence of the barrier (pilot data indicated that children often assumed towers covered by the barrier were no longer part of the game). To further emphasize this, E1 pointed to each platform on both sides at the beginning of each training section while saying "You can pull here, here, here, and here."

Follow-up preference tests

Apparatus understanding

All towers were baited and C collapsed one of the platforms and collected the rewards on three trials while the puppet sat passively in the responder position. To ensure that C experienced a similar number of trials as in Study 2, C and the puppet then switched positions and the puppet collect the marbles on three trials.

Barrier experience

A barrier spanning the whole apparatus blocked C's view of the towers (C was in the responder position). All towers were baited and the puppet collected the rewards on two trials. The puppet and C then swapped positions and C collected the rewards on two trials.

Statistical analyses

a) General information

For the main analyses we used Generalized Linear Mixed Models (GLMM) as this allowed us to simultaneously test for multiple predictors (and thus avoid multiple testing issues) while controlling for the influence of several potentially confounding variables. Moreover, since the chimpanzees came from two different social groups this analytic approach allowed us to include the social group as a random effect in order to avoid potential problems related to pseudoreplication. Finally, in Study 1b the GLMM enabled us to test for an interaction between the condition and the experimental phase which would not have been possible with standard non-parametric tests (e.g. Wilcoxon Signed Rank Test). The first GLMM in each study served to detect any condition differences to provide a first indicator for whether they successfully adjusted their choices to the social context. This analysis was followed up by a second GLMM looking at whether subjects made more correct choices in one of the conditions. All analyses were determined prior to data inspection.

b) Study 1 – Apes

Table S1 – Model descriptions

Model 1a – Did the condition have an effect on the apes' tendency to make their decisions visible? (Phase 1 only)
Model 1b – Did the condition, experimental phase or the interaction between condition and phase have an effect on the apes' tendency to make their decisions visible? (all data combined)
Model 2a – Did the condition and/or species have an effect on the apes' decision accuracy? (Phase 1 only)
Model 2b – Did the condition, experimental phase, the interaction between condition and phase, or species have an effect on decision accuracy? (all data combined)

Model	Dependent measure	Test predictor(s)	Control predictors	Random intercepts	Random slopes	Model type	Model diagnostics
1a	Choice visibility (visible vs. hidden)	Condition	Species^z, Trial number^z, Session number, Barrier position	Subject ID, Social group	Trial number, Session number, & Barrier position within Subject ID	GLMM	Overdispersion, Variance inflation factors (VIFs), Checks for influential cases
1b		Condition* Experiment phase					
2a	Choice accuracy (accurate vs. inaccurate)	Condition, Species	Trial number^z, Session number, Barrier position	Subject ID, Social group	Trial number, Session number, & Barrier position within Subject ID	GLMM	Overdispersion, VIFs, Checks for influential cases
2b		Condition* Experiment phase, Species					

^{z} Predictor was z-transformed
*denotes the interaction between two predictors, implies the inclusion of the main effects

Table S2 – Detailed model outputs

Model	Predictor	Estimate	Standard Error	X^2	DF	*p*
1a	Full-null model comparison			4.44	1	0.035
(Choice	Intercept	-0.064	0.387			
visibility,	Condition (cooperation)$^{\#}$	0.937	0.428	4.44	1	0.035
phase 1	Species (chimpanzees)^	0.229	0.412	0.305	1	0.581
only)	Barrier position	-0.101	0.387	0.065	1	0.799
	Trial^z	0.039	0.269	0.021	1	0.886
	Session^z	-0.012	0.167	0.005	1	0.943
1b	Full-null model comparison			10.72	3	0.013
(Choice	Intercept	-0.174	0.367			
visibility,	Condition*Phase^z	-0.043	0.299	0.021	1	0.885
all data)	Condition (cooperation)$^{\#}$	0.555	0.185	9.11	1	0.003
	Phase^z (2)$^+$	0.211	0.184	1.311	1	0.252
	Species (chimpanzees)^	0.434	0.279	2.212	1	0.137
	Barrier position	-0.332	0.297	1.178	1	0.278
	Trial^z	0.054	0.161	0.11	1	0.740
	Session^z	0.096	0.102	0.866	1	0.352
2a	Full-null model comparison			9.85	2	0.007
(Choice	Intercept	-0.765	0.35			
accuracy,	Condition (cooperation)$^{\#}$	1.008	0.337	7.137	1	0.008
phase 1	Species (chimpanzees)^	0.799	0.359	4.214	1	0.040
only)	Barrier position	0.685	0.322	4.534	1	0.033
	Trial^z	-0.257	0.249	1.019	1	0.313
	Session^z	0.108	0.172	0.391	1	0.532
2b	Full-null model comparison			47.47	4	< 0.001
(Choice	Intercept	0.174	0.367			
accuracy,	Condition*Phase^z	0.658	0.508	1.589	1	0.207
all data)	Condition (cooperation)$^{\#}$	1.12	0.188	37.396	1	< 0.001
	Phase^z (2)$^+$	-0.565	0.187	8.473	1	0.004
	Species (chimpanzees)^	0.132	0.285	0.212	1	0.645
	Barrier position	-0.056	0.313	0.032	1	0.859
	Trial^z	-0.238	0.133	2.857	1	0.091
	Session^z	0.077	0.092	0.702	1	0.402

^{z} Predictor was z-transformed

*denotes the interaction between two predictors, implies the inclusion of the main effects

$^{\#}$with the competition condition as the reference category

$^+$with phase 1 as the reference category

^with bonobos as the reference category

c) Study 2 – Children

Table S3 – Model descriptions

Model 3 – Did the condition have an effect on children's tendency to make their decisions visible?
Model 4 – Did the condition and/or species have an effect on children's decision accuracy?
Model 5 – Did condition or the number of accurate test decisions have an effect on children's responses to post-test question 1? ("Where was it easier to win the marbles?")
Model 6 – Did condition or the number of accurate test decisions have an effect on children's responses to post-test question 2? ("Why was it easier to win the marbles there?")

Model	Dependent measure	Test predictor(s)	Control predictors	Random intercepts	Random slopes	Model type	Model diagnostics
3	Choice visibility (visible vs. hidden)	Condition	Trial number[z], Barrier position, 2nd Experimenter	Subject ID	Trial number & Barrier position within Subject ID	GLMM	Overdispersion, VIFs, Checks for influential cases
4	Choice accuracy (accurate vs. inaccurate)	Condition	Trial number[z], Barrier position, 2nd Experimenter	Subject ID	Trial number & Barrier position within Subject ID	GLMM	Overdispersion, VIFs, Checks for influential cases
5	Response accuracy (correct vs. incorrect)	Condition, Number of accurate test choices	–	–	–	GLM	Overdispersion, Checks for influential cases, Absence of collinarity
6	Response accuracy (referring to partner's mental state or not)	Condition, Number of accurate test choices	–	–	–	GLM	Overdispersion, Checks for influential cases, Absence of collinarity

[z]Predictor was z-transformed

Table S4 – Detailed model outputs

Model	Predictor	Estimate	Standard Error	X^2	DF	p
3	Full-null model comparison			9.37	1	0.002
(Choice	Intercept	-1.617	0.598			
visibility)	Condition (cooperation)#	1.886	0.663	9.37	1	0.002
	E2	-0.663	0.622	1.12	1	0.290
	Barrier position	0.62	0.553	1.16	1	0.282
	Trial^z	-0.28	0.228	1.57	1	0.210
4	Full-null model comparison			9.61	1	0.002
(Choice	Intercept	2.257	0.642			
accuracy)	Condition (cooperation)#	-1.907	0.674	9.61	1	0.002
	E2	-0.706	0.613	1.34	1	0.247
	Barrier position	0.125	0.657	0.04	1	0.848
	Trial^z	-0.347	0.227	2.47	1	0.116
5	Full-null model comparison			7.95	2	0.019
(Question 1)	Intercept	-1.788	0.975			
	Number of accurate choices	0.423	0.205	4.71	1	0.030
	Condition (cooperation)#	1.657	0.732	5.86	1	0.015
6	Full-null model comparison			13.13	2	0.001
(Question 2)	Intercept	-3.779	1.284			
	Number of accurate choices	0.777	0.255	12.77	1	< 0.001
	Condition (cooperation)#	0.391	0.733	0.290	1	0.590

^{z}Predictor was z-transformed

#with the competition condition as the reference categor

Table S5 – Ape subject overview

Name	Species	Group	Age at start of study	Sex	First Condition	Competition: Partner	Competition: *N* Hidden Decisions	Cooperation: Partner	Cooperation: *N* Hidden Decisions	Preference Test: Partner	Preference Test: *N* Hidden Decisions
Frodo	Chimpanzee	a	22	m	Competition	Sandra	16	Kofi	12	Sandra	9
Lobo	Chimpanzee	a	12	m	Competition	Sandra	8	Sandra	10	Sandra	14
Tai	Chimpanzee	a	14	f	Competition	Sandra	15	Kofi	3	Sandra	13
Alex	Chimpanzee	b	15	m	Competition	Jahaga	16	Jahaga	8	Jahaga	6
Lexi	Bonobo	c	16	f	Competition	Fimi	8	Fimi	7	NA	*NA*
Luiza	Bonobo	c	11	f	Competition	Fimi	12	Fimi	11	Lexi	13
Kara	Chimpanzee	a	11	f	Cooperation	Sandra	13	Kofi	5	NA	*NA*
Kofi*	Chimpanzee	a	11	m	Cooperation	Sandra	8	Sandra	2	Sandra	12
Lome	Chimpanzee	a	15	m	Cooperation	Sandra	6	Kofi	7	Sandra	12
Alexandra	Chimpanzee	b	17	f	Cooperation	Jahaga	3	Jahaga	8	Jahaga	12
Kuno	Bonobo	c	19	m	Cooperation	Fimi	17	Fimi	8	Lexi	11
Yasa	Bonobo	c	19	f	Cooperation	Fimi	5	Fimi	12	Lexi	13
Bangolo	Chimpanzee	a	7	m	*NA*	*NA*	*NA*	*NA*	*NA*	Sandra	16
Corrie	Chimpanzee	a	39	f	*NA*	*NA*	*NA*	*NA*	*NA*	Sandra	12
Dorien	Chimpanzee	a	35	f	*NA*	*NA*	*NA*	*NA*	*NA*	Sandra	13
Natascha	Chimpanzee	a	36	f	*NA*	*NA*	*NA*	*NA*	*NA*	Sandra	11
Riet	Chimpanzee	a	38	f	*NA*	*NA*	*NA*	*NA*	*NA*	Sandra	11
Robert	Chimpanzee	a	40	m	*NA*	*NA*	*NA*	*NA*	*NA*	Sandra	8
Swela	Chimpanzee	a	20	f	*NA*	*NA*	*NA*	*NA*	*NA*	Sandra	12
Daza	Chimpanzee	b	30	f	*NA*	*NA*	*NA*	*NA*	*NA*	Jahaga	5
Frederike	Chimpanzee	b	42	f	*NA*	*NA*	*NA*	*NA*	*NA*	Jahaga	15
Gemena	Bonobo	c	10	f	*NA*	*NA*	*NA*	*NA*	*NA*	Lexi	11
Sandra	Chimpanzee	a	23	f				*Stooge partner*			
Fimi	Bonobo	c	8	f				*Stooge partner*			
Jahaga	Chimpanzee	b	23	f				*Stooge partner*			

*Also acted as stooge for some individuals of group a in Study 1B (after having completed the test himself) because the original stooge stopped participating. Stooge ID was included as a random effect in the analysis.
